# Cutaneous Manifestations of *Mycobacterium tuberculosis*: A Literature Review

**DOI:** 10.3390/pathogens12070920

**Published:** 2023-07-08

**Authors:** Kevin H. Nguyen, Cheldon Ann Alcantara, Ira Glassman, Nicole May, Akaash Mundra, Abinanda Mukundan, Bianca Urness, Sonyeol Yoon, Roajhaan Sakaki, Surbi Dayal, Tanzila Chowdhury, Shakila Harshavardhan, Vadakupattu Ramanathan, Vishwanath Venketaraman

**Affiliations:** 1 Department of Basic Sciences, College of Osteopathic Medicine of the Pacific, Western University of Health Sciences, Pomona, CA 91766, USA; kevin.nguyen2@westernu.edu (K.H.N.); cheldonann.alcantara@westernu.edu (C.A.A.); ira.glassman@westernu.edu (I.G.); nicole.may@westernu.edu (N.M.); akaash.mundra@westernu.edu (A.M.); abinanda.mukundan@westernu.edu (A.M.); bianca.urness@westernu.edu (B.U.); sonyeol.yoon@westernu.edu (S.Y.); roajhaan.sakaki@westernu.edu (R.S.); surbi.dayal@westernu.edu (S.D.); tanzila.chowdhury@westernu.edu (T.C.); 2 Department of Molecular Microbiology, Madurai Kamaraj University, Tamil Nadu 625021, India; mohanshakila.biotech@mkuniversity.ac.in; 3 Department of Pathology, National Institute for Research in Tuberculosis, Chennai 600031, India; vdrnathan@yahoo.com

**Keywords:** tuberculosis, skin, infectious disease, dermatology

## Abstract

Tuberculosis is an ancient disease that humanity struggled with for centuries and continues to struggle with. The bacteria *Mycobacterium tuberculosis* often infects the lungs through respiratory transmission and manifests itself through various symptoms, including cutaneous infections. Cutaneous tuberculosis (CTB) comprises about 1% to 1.5% of all extrapulmonary manifestations and is often accompanied by polymorphous lesions, including papules, nodules, plaques, ulcers, gummas, and verrucous lesions. CTB is most commonly observed in low-income, HIV, and immunosuppressed populations, similar to intrapulmonary manifestations. The main pathogen for CTB is *M. tuberculosis* but less commonly with *M. bovis* and BCG vaccine, and the modes of transmission are largely classified into exogenous and endogenous CTB. Current treatment options for CTB include oral therapy of antibiotic medications such as rifampicin, streptomycin, ethambutol, isoniazid, and pyrazinamide, which is occasionally combined with surgical intervention.

## 1. Introduction

Mycobacterium tuberculosis (*M. tuberculosis*) is the bacterium responsible for the contagious and infectious disease tuberculosis (TB). The more uncommon form of this infectious disease, cutaneous tuberculosis (CTB), accounting for 1 to 1.5% of all extrapulmonary TB manifestations, is prevalent only in 8.4–13.7% of all TB cases [1]. CTB can be further classified by infection from *M. tuberculosis*, known as true CTB, or from an atypical mycobacterium species [1]. *M*. *tuberculosis* can be described as a straight, unencapsulated, rod-shaped bacillus that is not sporulated and lacks features for motility; its size is typically between 1 to 10 μm in length and 0.2 to 0.6 μm in width.

How the disease clinically presents is variable due to external factors such as the host’s immunity, the site of the infection, or even the surrounding environment of the host [2]. The clinical manifestations of CTB can vary from inflammatory papules, verrucous plaques, suppurative nodules, and chronic ulcers to painless, isolated, purplish, or brownish-red warty plaques that can discharge pus or other keratinous material [2].

The pathogenesis of CTB initially forms after pulmonary infections, beginning from the inhalation of the bacterium into the alveoli, which activates the alveolar macrophages in response to eliminating the bacillus. Once the *M. tuberculosis* bacterium overcomes the immunological response, it then begins to divide within the macrophages, cause tissue damage and destruction, and spread via the bloodstream circulation to reach the dermis. 

Current treatment options for CTB include, but are not limited to, oral therapy and occasional surgical intervention or a combination of rifampicin, ethambutol, pyrazinamide, isoniazid, and streptomycin, as well as utilizing a two-phase treatment plan [1]. Nevertheless, those with CTB caused by atypical mycobacterium species were found to be more drug-resistant. Currently, there are no topical treatment solutions for CTB, therefore justifying the necessity for further research into alternative treatment options [1].

In this review, we aim to summarize the pathogenesis, risk factors, and treatment options for CTB. Despite current and recent advancements in technological treatments, CTB continues to be a pressing global health concern in developing countries and, therefore, needs to be further researched to offer more effective alternatives.

## 2. Incidence and Epidemiology of TB and Cutaneous TB

According to the World Health Organization (WHO), about 10.6 million people were infected with *M. tuberculosis* in 2021 globally, with the majority of incidences from South East Asia (45%), Africa (23%), and Western Pacific (18%). Five countries with the highest TB incidences were: India (28%), Indonesia (9.2%), China (7.4%), Philippines (7.0%), and Pakistan (5.8%) [3]. In 2020, 10.1 million cases were reported, which was a result of a declining trend of about 2% per year in the past 20 years [3]. The reversal of the decline trend with a 4.5% increase in number of cases from 2020 to 2021 has been attributed to the surge of COVID-19 in 2019, impacting major health facilities [3]. Reduced detection of new TB cases from the impact may have possibly led to reduced diagnosis and treatment, accruing consequences to be manifested in 2021.

There are numerous risk factors that may contribute to the development of TB, including lack of a hygienic environment, homelessness, overcrowding, substance abuse, alcohol abuse, cigarette smoking, HIV, diabetes mellitus, immunosuppressive therapy, and malnutrition [4]. According to Mehta and Dutt, there was a 20% increase in TB cases in the United States between 1985 and 1992, and most of the cases arose within cities with populations greater than 500,000, especially among the poor, including the homeless [5]. Furthermore, the rate of incidence was approximately eight times higher in those with the lowest median income compared to those with the highest median income. This phenomenon was also supported by WHO, as over 80% of cases and deaths arise in low- and middle-income countries, such as South Africa, Democratic Republic of Congo, and Ethiopia. Reduction in immune function has also been strongly implicated as a risk factor for TB, as immunocompromised populations are disproportionately affected. HIV, therefore, serves as a strong risk factor. According to WHO, the likelihood of developing TB in the HIV population is about 16 times higher than those without. People with HIV constituted 6.7% of all TB cases in 2021, with the highest number of cases in the African region, such as South Africa, Mozambique, and Zimbabwe [3]. In 2020, six countries, including Malawi, Uganda, Tanzania, Kenya, Nigeria, and Ethiopia, in addition to the three aforementioned African countries, accounted for approximately 72% of all new TB cases among people living with HIV in the African region [3].

The epidemiology of CTB is not well studied; however, its incidence tends to occur in countries endemic to *M. tuberculosis*. With an increase in the incidence of TB over recent years, the emergence of new cases of CTB has similarly occurred [6,7,8]. Several reports of CTB have been studied in countries endemic to TB, such as Tunisia and Morocco [9,10]. Many parts of India, including Chandigarh, Varanshi, Chennai, and Delhi, report an increased incidence of immunocompromised children [11,12,13,14]. Furthermore, CTB had a relatively large prevalence in Hong Kong in the past; however, recent surveys suggest otherwise [15].

The importance of immune function is implicated in CTB. In a study conducted by Lotte et al., a group of 5000 patients who experienced adverse reactions to the BCG vaccine were assessed, and 28 were diagnosed with disseminated mycobacterial disease [16]. Of those 28 patients, 24 had some form of immunodeficiency, while 9 were also diagnosed with acquired immunodeficiency syndrome (AIDS). The possibility of disseminated mycobacterial infection secondary to BCG vaccination has also been observed. According to a case study by Dhar et al., the administration of the BCG vaccine first started as a local swelling of the injection site, which then resulted in the disseminated mycobacterial infection in an immunocompetent child [17]. Dhar notes the rarity of the dissemination of mycobacteria from the BCG vaccine because local cutaneous complications, along with other CTBs such as lupus vulgaris, tuberculids, and even leprosy, are not uncommon [1]. Since the development of CTB is in part contributed by the immune function of the host as well as direct inoculation, the risk factors for cutaneous disease include HIV/AIDS, dermal piercing, tattoos, mesotherapy, acupuncture, intravascular devices, postsurgical infections, freshwater or saltwater injuries, chronic pulmonary diseases, and renal transplant [18]. Particularly, regions with a high prevalence of HIV and pulmonary TB are observed to witness a rise in CTB [19]. 

## 3. Molecular and Cellular Background of CTB

CTB may have a distinct molecular and cellular presentation compared to pulmonary TB, particularly regarding spoligotypes, resistotypes, animal models, and antigens. Cultures are elaborated from CTB; therefore, it is typically in the active phase [20]. *M. tuberculosis* complex includes *M. tuberculosis*, *M. bovis*, *M. africanum*, *M. microti*, *M. canetti*, *M. caprae*, and *M. pennipedii* [21]. Multiplex PCR can easily distinguish between strains of *M. bovis*, including BCG-related *M. bovis* strains, from those that are unrelated to the vaccine. TCH susceptibility distinguishes *M. tuberculosis* (resistant) from *M. bovis* (susceptible) [22].

Sibandze et al. investigated spoligotypes involved in extrapulmonary tuberculosis infections in South Africa, a region with one of the highest tuberculosis disease burdens [23]. They cultured isolates and clustered M. tb strains into four lineages: East Asian (1 case), East African (1 case), Euro-American (5 cases), and an orphan lineage (3 cases), which was considered nonclustered due to unmatched genetic strains. They found that cutaneous, lymphadenitis, and meningitis cases were significantly more likely to be associated with drug resistance, with 45% of sampled cutaneous TB cases demonstrating pan-resistance and 63% of cases with Rifampin and/or MDR-TB resistance. They also found that there was no association between drug resistance and spoligotypes for any resistance [23].

In China, Mei et al. investigated nine lineages of *M. tuberculosis* and found that the Beijing family and Euro-American lineages were the most prevalent lineages in China [24]. Their research differed from the data in South Africa in that cutaneous tuberculosis was found to be less resistant than pulmonary tuberculosis in adjacent provinces, with 12.07% of cutaneous tuberculosis possessing resistance. This was explained by a lower drug mutation rate and differences in SNPs of metabolic genes in cutaneous TB isolates [24].

Mice, guinea pigs, and rabbits are the most used animal models for the study of TB [25]. When infected with virulent *M. tuberculosis*, rabbits usually recover, similar to how most humans recover, whereas guinea pigs and mice infected with virulent *M. tuberculosis* usually die [26]. In a discussion on animal models, Zhan et al. assert that the New Zealand rabbit and Chinese tree shrew may be utilized for CTB research [27].

The similarity in pathology between the spectrum of rabbit and human TB has been recognized for decades [28]. Zhang et al. found that intradermal BCG induces healing tuberculous lesions in the New Zealand white rabbits [29]. Skin lesions were removed and subjected to acid-fast staining by the Ziehl–Neelsen method. A sufficient number of mycobacteria is required to induce lesions in the rabbit skin model, as low doses (5 × 10^2^ CFU) were not found to induce obvious lesions compared to high doses (5 × 10^6^ CFU). This indicates that CTB lesions with a high bacillary load, including disseminated miliary TB, scrofuloderma, and gummatous TB, may be studied using this model. 

Chinese tree shrews have been found to form CTB lesions when infected with *M. tuberculosis*, which have not been seen in other animal models like monkeys or mice [30]. Zhan et al. infected Chinese tree shrews with a high dose (2.5 × 10^6^ CFU) or a low dose (2.5 × 10^3^ CFU) of the H37Rv strain. This suggests that the Chinese tree shrew has greater potential for serving as a CTB animal model. Other advantages of this model include ease of availability, good cost-effectiveness, and easy maintenance [30]. Both the rabbit skin model and the Chinese tree shrew model show potential for CTB research, yet further studies are needed to confirm this.

Chen et al. propose that proteins are the most important antigens of *M. tuberculosis* [31]. Serology testing for *M. tuberculosis* is typically carried out with a highly purified series of antigens, including the proteins Ag-60, 30 kDa, 38 kDa, 45/47-kDa complex, and Kp90 or the antigens DPEP, MPT32, Mtb81, and ESAT-6 [32,33,34,35,36]. Combining antigens in the same test can increase sensitivity with a subsequent decrease in specificity. Since many cases of CTB seem to be correlated with pulmonary TB, antigens that are expressed throughout each disease may be similar. However, the applicability in cases of extrapulmonary TB, namely CTB, remains unknown [37]. The application of next-generation transcriptomics, LC/MS analysis, and high-throughput real-time qPCR for the assessment of gene patterns of each type of CTB clinical manifestation would permit the identification of biomarkers that would provide insight into pathogenesis and specific treatment plans. Similarly, this would allow the comparison of CTB lesions between New Zealand white rabbits, Chinese tree shrews, and humans to test the utility of animal models for CTB research. Overall, more research is needed to study the similarities and differences between CTB and pulmonary TB. 

## 4. Pathogenesis of CTB

CTB classification, discussed further in the next section, is based on the route of propagation: exogenous, endogenous, or hematogenous. Exogenous infection occurs from direct contact on the skin, while endogenous infection is when cutaneous involvement occurs secondarily by contiguity from an already established focus, while a hematogenous route can occur from a distant location [38]. The skin manifestations vary in presentation depending on the pathogenicity of the bacterial strain, the immune status of the infected individual, and local factors such as proximity to lymph nodes [39]. CTB is often seen histologically as a granuloma formation, but TB can also cause vasculitis and panniculitis [40].

**Figure 1:**
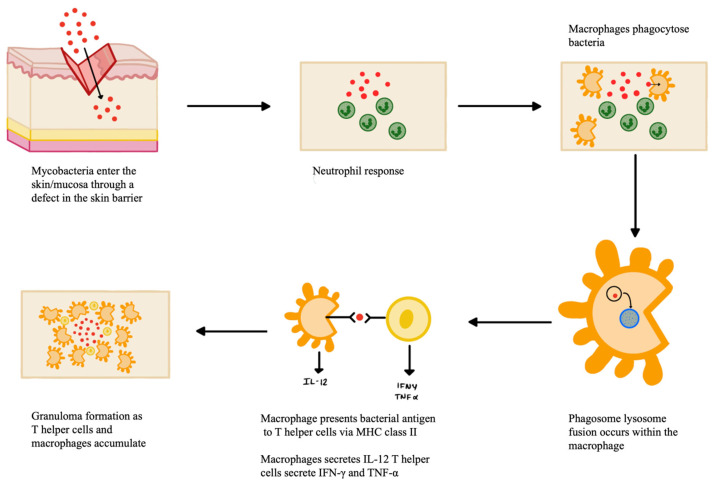
Pathogenesis of *M. tuberculosis*.

Primary inoculation of TB from an exogenous source occurs when mycobacteria enter the skin or mucosa of a person with no immunity to *M. tuberculosis* through a defect in the skin barrier, such as an abrasion or wound, represented in Figure 1 below. The organism multiplies within the skin as the body’s immunological response begins [6]. The initial immune response is dominated by neutrophils, followed shortly after by natural killer cells and macrophages. The neutrophils recognize and phagocytose bacteria via antibody Fc receptors and complement system activation receptors [38]. This neutrophil response creates an area of necrotizing inflammatory infiltrate, and tubercule bacilli are still seen early in the infection [40]. Macrophages also make up one of the first lines of defense against TB. The macrophages phagocytose the bacteria, where phagosome lysosome fusion takes place. The bacteria antigens are processed, and macrophages present them to T helper lymphocyte cells (CD4+) via the major histocompatibility complex (MCH) class II. Apoptotic vesicles that come from infected cells also contain antigens and can specifically stimulate CD8+ T cells. Macrophages also secrete IL-12, which induces the production of IFN-γ by Th1-cells. IFN-γ is an important mediator in the macrophage activity against *M. tuberculosis* as it increases MHC expression to allow for antigen presentation, recruits T lymphocytes, and increases expression of immunoglobulin receptors [38]. Over the next few weeks, these macrophages accumulate to form a granuloma around the area of inoculation to wall off bacteria and prevent their further spread [6]. The macrophages within the granuloma have a high turnover rate and include multiple phenotypes of macrophage, including epithelioid cells, multinucleated giant cells, and foamy cells [41]. Granuloma formation is mediated by TNF-α, a proinflammatory cytokine secreted by T lymphocytes, monocytes, and macrophages. TNF-α maintains granuloma structure by increasing adhesion molecule expression and produces reactive oxygen and nitrogen intermediates [42]. TNF-α also regulates the synthesis of IL-12 and NF-kB, which help to expand the CD4+ Th1 lineage to allow for more comprehensive antigen presentation [38]. The granuloma will persist as long as Th1 helper cells respond to *M. tuberculosis* antigens, leading to continuous phagocyte activation and inflammation to limit the dissemination of bacteria [41]. On gross examination of the inoculation site, a nodule first erupts that eventually develops into a firm, non-tender, sharply delineated ulcer known as a tuberculous chancre [40]. Over the next few weeks, the infection can extend to the lymphatic system causing lymphadenopathy and, eventually, draining nodules with calcification. If a person has previous TB immunity, the cutaneous inoculation may result instead in a hyperkeratotic papule that becomes verrucous [6]. This type of asymptomatic warty lesion is called a TB verrucosa cutis, and it frequently spontaneously resolves over the next months to years [40]. Lymphadenopathy or ulceration is rarely seen in these cases [6].

**Figure 2:**
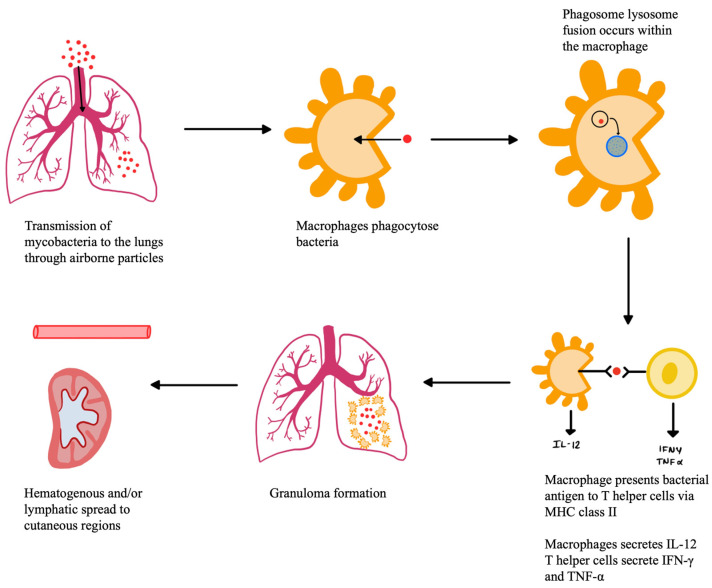
Hematogenous Spread of *M. tuberculosis*.

An endogenous inoculation of TB can occur by hematogenous, lymphatic, or contiguous spread, originating from a primary site of infection, represented below in Figure 2. Hematogenous and lymphatic seeding may cause metastatic tuberculous abscesses, also known as tuberculous gummas, especially in individuals who are immunocompromised. These gummas are often located on the trunk and extremities, or they can be found along the lymphatics. Another form of CTB caused by hematogenous or lymphatic spread is lupus vulgaris. Lupus vulgaris is a cutaneous lesion histologically made up of granulomas with caseating necrosis in the upper dermis. On gross examination, these lesions appear as a yellow-reddish cutaneous plaque with desquamation and central atrophy, often appearing in the facial and cervical areas [39,40]. Contiguous inoculation occurs on a location overlying a primary TB focus, such as a lymph node, bone, or epididymitis. Initially, a firm, mobile nodule can be palpated, which eventually attaches to the overlying skin, suppurates, and forms a draining cutaneous abscess or sinus tract. On histology, the caseation necrosis and granuloma are slowly replaced by chronic inflammatory cells over time as the lesions heal [6]. Scrofuloderma occurs when there is an underlying tuberculous infection, such as a lymph node, gland, testicle, or bone, that spreads contiguously to create a cold abscess on the overlying skin. These lesions appear as painless purple nodules with histological caseating granulomatous inflammation in the lower dermis [39,40]. Orofacial TB occurs when acid-fast bacteria are shed from the primary foci and inoculated into mucocutaneous areas, such as oral, nasal, anal, or vulval mucosa [6]. This causes painful ulcerative lesions of the mucosa that appears as a yellow papule, with histological granuloma and caseation necrosis formation. Orofacial TB often impacts individuals with advanced TB, already affecting other organs and who have impaired cell-mediated immunity [40]. 

## 5. Clinical Presentation of CTB

CTB is often overlooked due to its rare occurrence as well as its wide range of possible morphologies. A diagnosis of CTB requires a high index of suspicion in addition to histopathology, culture, and PCR. About one-third of CTB cases present with systemic involvement [43]. CTB manifestations are typically categorized based on the mechanism of propagation: exogenous, endogenous, or hematogenous [2,6,19,44,45]. Classifications may include host immune status, previous treatment, or bacterial load. The classification for TB based on the mechanism of propagation will be adopted in this work. Exogenous CTB includes tuberculous chancre and TB verrucosa cutis [2]. Endogenous CTB includes scrofuloderma and TB orificialis. Hematogenous spread causing CTB includes lupus vulgaris, metastatic tuberculous abscess, and acute miliary TB [46]. Physicians should have a low threshold for considering CTB as a differential diagnosis upon presentation of a cutaneous lesion with any of the following descriptions.

**Figure 3:**
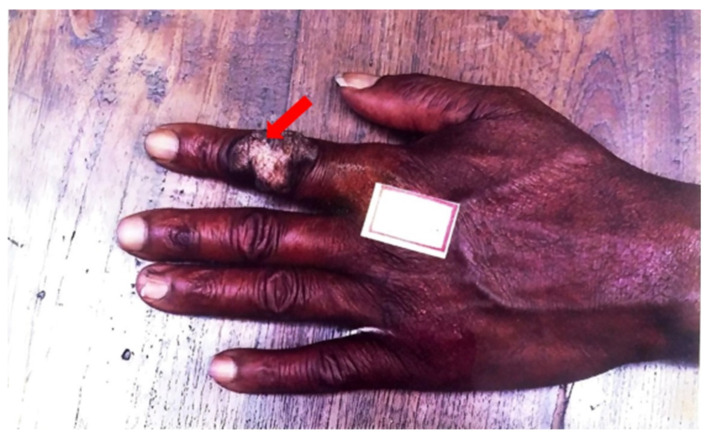
TB verrucose cutis at presentation.

Exogenous primary inoculation of CTB has been shown to manifest through the entry of *M. tuberculosis* into skin and mucosa, resulting in a tuberculous chancre or TB verrucosa cutis, represented in Figure 3 below. Analogous to how the Ghon complex is a key finding of primary pulmonary TB, the complex of the tuberculous chancre and regional adenopathy may serve as the cutaneous counterpart [6]. These lesions typically occur after a local trauma, including surgical procedures with unsterilized materials or tattoos. A tuberculous chancre initially presents as a reddish-brown firm and nontender papulonodular lesion accompanied by lymph node enlargement. The lesion rapidly grows, then erodes to result in a painless, well-demarcated ulceration with a coarse granular base and red, blue, and undermined edges. These lesions persist for many years but may spontaneously regress without therapy to leave behind atrophic scars or progress into a lupus vulgaris-like lesion [19,44]. Post-primary, or secondary, inoculation of exogenous CTB manifests by spreading the disease from the lungs to the skin and mucosa. TB verrucosa cutis, otherwise known as post-primary inoculation CTB, presents as a solitary, slow-growing red-brown papule with no lymphadenopathy. It enlarges to form a verrucous plaque resembling a common wart with clefts and fissures on its surface. It usually manifests on an extremity, such as the hand or the foot [47]. The development of multiple tender or non-tender ulcers, papules, and warts are characteristic of progression [6]. Progression of both primary and post-primary CTB infections is variable and dependent on patient age, immune response, and other similar factors.

**Figure 4:**
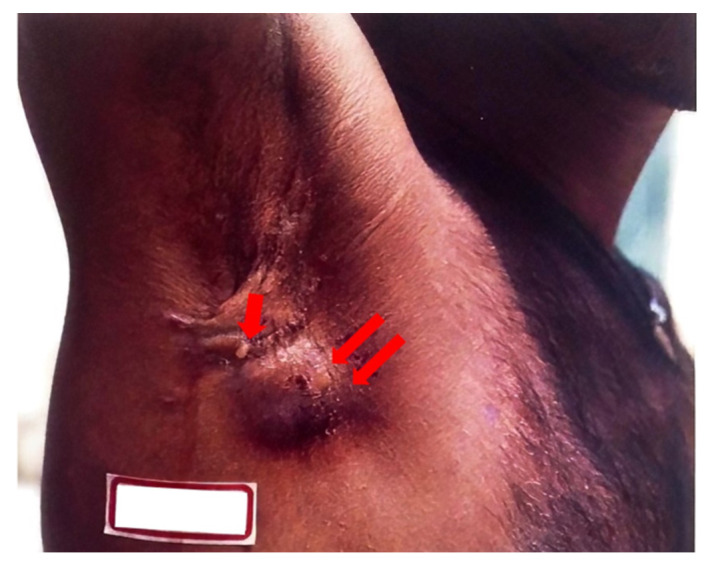
Scrofuloderma at presentation. Axillary lymph node (double arrow) with discharging sinus (single arrow).

Lesions of endogenous CTB infections result from the breakdown of the skin covering a subcutaneous focus of *M. tuberculosis.* These are characterized as scrofuloderma or orofacial TB. A scrofuloderma, shown in Figure 4 below, presents with cutaneous symptoms due to the spread of the disease from lymph nodes to skin, bones, and joints. Scrofuloderma starts as a painless, red-brown firm nodule or swelling overlying an affected lymph node that becomes indurated and breaks down to form a discharging sinus tract with watery, purulent, or caseous material. The initial presentation of a painless, subcutaneous nodule can also be referred to as a cold abscess [48]. Typically, these lesions appear at prominent lymph node sites, including the neck, axillae, chest wall, and groin [44]. Kim et al. describe a case where cutaneous TB, presenting as a scrofuloderma, was correctly diagnosed only after antibiotics had not successfully eradicated what was originally thought to be a bacterial abscess [48]. A retrospective study in Brazil found that scrofuloderma represented 50.7% of CTB cases [49]. Orofacial TB results from the autoinoculation of the mucous membrane, manifesting on the lips, inside the mouth, or on the anogenital area as a painful ulcer with a pseudomembranous fibrinous base [44]. Patients with these lesions are likely to have progressive pulmonary, genital, urinary, or intestinal TB.

**Figure 5:**
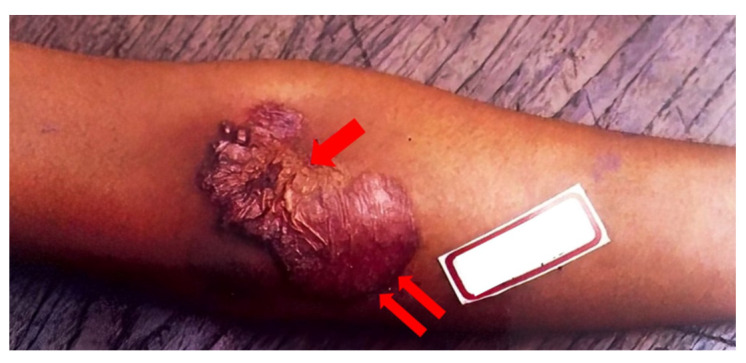
Lupus vulgaris at presentation. Single arrow shows the healing periphery, while the double arrow shows the active edge.

Hematogenous-spread CTB includes lupus vulgaris, metastatic tuberculous abscess, and acute miliary TB [46]. These lesions occur when *M. tuberculosis* spreads from a primary site of infection to the rest of the body. Malignancy, disfiguration, and Hodgkin’s Lymphoma are conditions associated with hematogenous CTB infections [6]. The most common form is lupus vulgaris, depicted below in Figure 5 [1]. Lupus vulgaris manifests on the face or neck as a group of small brown-red soft papules that coalesce into a gelatinous plaque. In the tropics and subtropics, the lower extremities and buttocks are most often involved. Upon diascopy, apple jelly nodules are usually found [45]. Two retrospective studies, one conducted in China and the other in central India, found lupus vulgaris to be the most common clinical manifestation of CTB at rates of 32.7% and 51.92%, respectively [50,51]. Metastatic tuberculous abscess, otherwise known as a tuberculous gumma, manifests as multiple, non-tender skin nodules on the trunk, extremities, or head that may ulcerate to form a discharging sinus tract. Eventually, these lesions may become indistinguishable from scrofuloderma [19]. TB miliaris cutis disseminate manifests on the trunk, thighs, buttocks, or genitalia as widespread erythematous macules or papules topped with minute vesicles that eventually rupture or dry. The lesion may heal within one to four weeks as a white depressed scar with a brownish halo [19].

Several case reports exist of cutaneous manifestations after the administration of the BCG vaccine. Most recipients of the BCG vaccine report a benign and localized skin reaction after administration, although there is a risk for more serious cutaneous side effects such as fistulation, abscess formation, and ulceration. Even rarer, a tuberculous chancre can form with inoculation of *M. bovis* to the skin after local trauma, and this accounts for about 1–2% of CTB [52]. Multiple reports have been published describing the extremely rare development of a CTB reaction after the administration of the BCG vaccination [17]. Dhar et al. describe a previously healthy 4-month-old diagnosed with biopsy-confirmed disseminated CTB at the site of BCG immunization that resolved without recurrence after anti-TB treatment [17]. A case report conducted by Stratmen et al. described an adult developing chancre-variant CTB in response to receiving the BCG vaccine for the treatment of fibromyalgia [52]. The patient underwent surgical debulking of the chancre as well as 6 months of Isoniazid monotherapy, and the complication resolved. Keijsers et al. also showed the development of a TB chancre in a male child after administration of the BCG vaccine that resolved following excisional biopsy [53]. There is a lack of knowledge regarding BCG-related cutaneous complications, and as the vaccine becomes increasingly popular in the United States as an off-label method for managing autoimmune and inflammatory conditions, an increased incidence of cutaneous manifestations of TB may be seen.

Due to the high nature of the variability of clinical findings of CTB, it is often recommended to proceed with a high degree of suspicion. Abscesses, nodules, plaques, and other lesions are seen in CTB and can provide a clue as to the type of CTB infection that is present, but it is difficult to confidently diagnose a specific condition without additional lab testing. The most common forms of CTB may vary depending on geographical location and patient demographics.

## 6. Histology of Cutaneous TB

The histology of CTB lesions somewhat resembles that of *M. tuberculosis* due to the presence of inflammation, necrosis, and acid-fast bacilli. Lesions differ with types of necrosis, types of immune cells present, and layers of the skin that are affected. 

**Figure 6:**
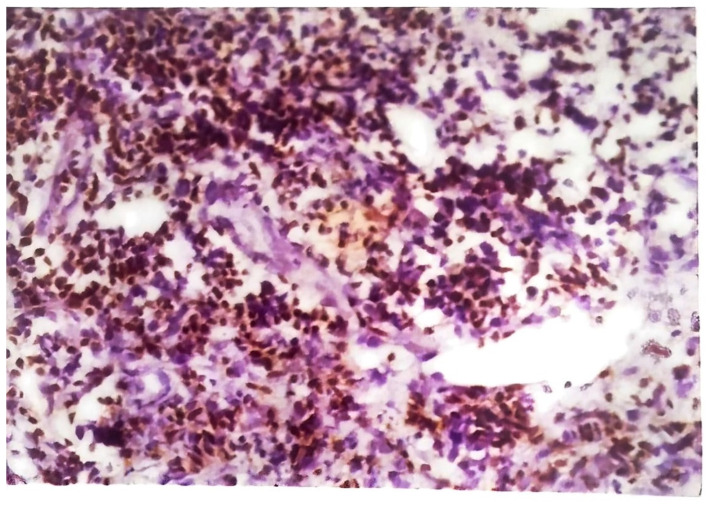
Clusters of T lymphocytes stained with anti-CD3 antibody seen in the lesion of tuberculous verrucose cutis (indirect immunoperoxidase method, magnification 1.67 × 40).

**Figure 7:**
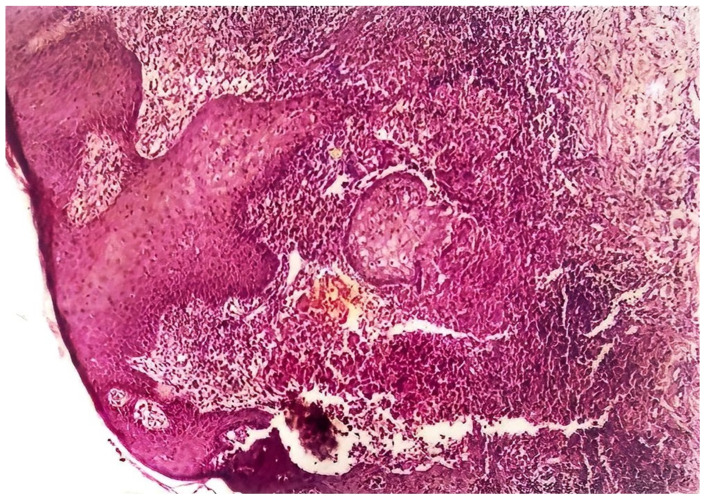
Section of lesion in Figure 5. Marked acanthosis, dermal granuloma consisting of epitheloid cells, lymphocytes, and giant cells. (H&E Magnification 1.67 × 40).

**Figure 8:**
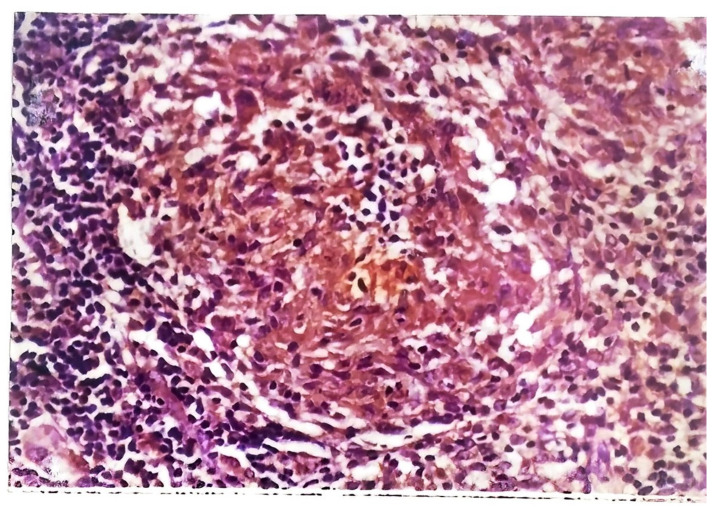
Granuloma from a case of lupus vulgaris. Section stained with anti-*M. tuberculosis* using indirect immunoperoxidase staining method. Note the brown stained cells of the mononuclear macrocytes series containing *M. tuberculosis* antigen (Magnification 1.67 × 40).

The histology of tuberculous chancres resembles that of an acute inflammatory reaction with areas of necrosis, numerous tubercle bacilli, and neutrophils [44]. After three to six weeks, the lesion involves granulomatous inflammation with increased numbers of giant cells and epithelioid cells and decreased number of identifiable tubercle bacilli. Healing may occur slowly with fibrosis and calcification [45]. Skin biopsy of tuberculous verrucosa cutis (Figure 6) shows hyperkeratosis, papillomatosis, and pseudoepitheliomatous hyperplasia of the epidermis as well as tuberculoid granulomas in the dermis. In about a third of patients with verrucosa cutis lesions, neutrophilic microabscesses were present in the epidermis [45,47]. Endogenous CTB infections, scrofuloderma, and orofacial TB involve granulomatous inflammation with caseous necrosis and acid-fast bacilli. The sinus tracts affected by scrofuloderma may exhibit predominantly nonspecific acute and chronic inflammation, with necrosis at the center. In the epidermis, extensive cicatricial bands may be found due to scarring and atrophic changes. Orofacial TB is characterized by nonspecific inflammation and necrosis in addition to poorly defined granulomata [45]. The histology of CTB lesions spread hematogenously includes the presence of acid-fast bacilli and inflammation as well. TB miliaris cutis involves nonspecific inflammation with necrotizing vasculitis and acid-fast bacilli. Metastatic tuberculous gumma involves granulomatous inflammation with caseous necrosis and acid-fast bacilli. Lupus vulgaris (Figure 7) causes nonspecific inflammation and may also show pseudoepitheliomatous hyperplasia, acanthosis, and hyperkeratosis of the epidermis. Umapathy et al. conducted a study with patients that had lupus vulgaris, scrofuloderma, or verruca cutis lesions [47]. The granuloma (Figure 8) in all three of these types of CTB consisted of epithelioid cells, macrophages, and Langerhans giant cells. Plasma cells were present in moderate numbers as well, except in verrucosa cutis. Caseating necrosis was present predominantly in scrofuloderma and rarely in lupus vulgaris or verrucosa cutis lesions. Bravo et al. also found that lupus vulgaris and verrucosa cutis lesions demonstrate noncaseating tuberculous granulomata [44]. Lupus vulgaris was observed largely over the extremities, verrucosa cutis in the sole and foot, and scrofuloderma over the lymph nodes [47].

## 7. CTB Treatments

Treatment options for CTB primarily follow systemic treatment regimens. Multi-drug treatments are commonly utilized, including isoniazid, rifampicin, pyrazinamide, and ethambutol or streptomycin [54]. Isoniazid alone has proven effective with high cure rates, but the supplemental use of chemotherapy is supported for individuals with significant immunosuppression or multiple skin lesions [6]. Selective growth of resistant bacterial mutants creates drug resistance of *M. tuberculosis*. The severity of the drug resistance is dependent on the number of bacilli and the likelihood of drug-resistant mutants in the lesion. When only one individual drug is used, the probability of drug-resistant mutants is 1 in 10-3–10-8. When two drugs are used, the probability becomes 10-12–10-14 for two drugs and 10-18–10-20 for three drugs. This creates the foundation for chemotherapy utilization for TB treatment [55]. 

The treatment of CTB involves two phases of treatment. The essential and first-line drugs utilized in TB are isoniazid, rifampin, ethambutol, pyrazinamide, and streptomycin. The second-line drugs include aminoglycosides (kanamycin, amikacin), quinolones (ciprofloxacin, ofloxacin, levofloxacin), ethionamide or prothionamide, cycloserine, para-aminosalicylic acid (PAS), and polypeptide (capreomycin). The utilization of both classes of drugs during the two-phased chemotherapy creates the foundation for the unique treatment of TB. The initial phase is a multi-drug, intensive phase designed to substantially reduce the bacillary load and number of drug-resistant organisms. Isoniazid kills around 95% of the organisms during the first two days. Rifampicin and pyrazinamide are used next in the initial intensive phase. The second phase, the continuation phase, is utilized to kill the drug-sensitive bacilli that became dormant during the initial phase [55]. The first phase lasts around 8 weeks. The second phase, the continuation phase, lasts between 9 and 12 months [54]. 

A surgical intervention provides further therapeutic effects in aiding with debridement and diagnosis. Debridement serves diagnostic purposes as it provides biopsies for skin lesions that may be tubercular in origin. These biopsies can be utilized for histopathological purposes. Enzyme-linked immunosorbent assays for antibodies to *M. tuberculosis* antigen 5, monoclonal antibody assays, and PCR techniques have become important clinically for rapid and accurate identification of *M. tuberculosis* in skin lesions [6]. 

In 2015, the use of topical treatments had yet to be identified or developed [1]. However, a case study was performed in 2017 that utilized topical application of anti-TB drugs for the treatment of erythema induratum, a chronic recurrent lobular panniculitis commonly associated with TB infection. More specifically, tuberculous granulomatous panniculitis without vasculitis is a rare presentation of CTB that requires the correlation of clinical findings with histopathology and microbiological tests to confirm the diagnosis [56]. In this study, 15 tablets of 1500 mg isoniazid were crushed and mixed with 40 g zinc oxide ointment, creating a 3.75% topical anti-TB cream. After a month of topical application of the treatment twice daily, the skin lesions showed improvement [57].

## 8. Testing for TB

Rapid and accurate CTB diagnosis and treatment lead to reduced morbidity and mortality. The gold standard for the diagnosis of CTB is a mycobacterial culture of skin biopsy specimens [2]. The development of PCR has allowed for the rapid and accurate identification of *M. tuberculosis* in vitro. More recently, genotyping has been shown to aid in CTB diagnosis as well. Given the broad spectrum of potential clinical manifestations and rarity of the disease, diagnosis of CTB requires a low threshold of suspicion. 

A skin biopsy is evaluated in two ways. First, slides are prepared from the cutaneous lesion and examined using a microscope to detect acid-fast bacilli. Second, the skin tissue is cultured to detect the growth of *M. tuberculosis* [58]. Acid-fast smears are helpful in the diagnosis of cutaneous lesions with high bacillary load, including disseminated miliary TB, scrofuloderma, and gummatous TB. The mycobacterial cell wall is rich in complex lipids, which stain red with a Ziehl–Neelsen stain and yellowish red with an auramine-rhodamine stain. This is not specific for *M. tuberculosis*, as *Nocardia*, *Corynebacterium*, nontuberculous mycobacteria, and even artifacts may exhibit acid-fast staining. There are lower sensitivities of staining in extrapulmonary infections compared to pulmonary TB infections. Depending on the form of CTB, the presence of acid-fast bacilli is variable [59]. Therefore, the most specific diagnostic method is a culture of *M. tuberculosis*. However, the slow growth of *M. tuberculosis* is a major limiting factor to quick diagnosis and treatment. If enough viable mycobacteria are present, cultures may take up to 8 weeks or longer to develop. Typically, the bacillary load in CTB is less than in pulmonary TB [19].

PCR can be used to identify *M. tuberculosis* in vitro, specifically the bacterial 16S ribosomal DNA. DNA present in skin biopsies undergoes denaturation, hybridization, elongation, and amplification to confirm the presence of mycobacteria. PCR has already been utilized to identify CTB in a wide variety of manifestations ranging from scrofuloderma to papulonecrotic tuberculids [19]. Khadka et al. found wide ranges of sensitivity and specificity in the diagnosis of CTB [59].

Genotyping distinguishes atypical mycobacteria from *M. tuberculosis* and detects drug resistance [59]. This is carried out by analyzing germline DNA. There are various methods of genotyping, including spoligotyping, Mycobacterial Interspersed Repetitive Unit-Variable Number Tandem Repeats (MIRU-VNTR), and Restriction Fragment Length Polymorphism (RFLP). This method is especially helpful in distinguishing CTB from extrapulmonary presentations of *M. bovis*.

Almost all the investigative methods have lower sensitivities and specificities for CTB diagnosis compared to that of pulmonary TB. Diagnosis is made using diagnostic test results, patient history, and patient clinical presentation. Currently, skin biopsy with subsequent acid-fast staining and cultures is the gold standard for CTB diagnosis. PCR and genotyping both provide another method for detection. Accurate diagnosis with subsequent management is ideal for controlling the spread and prevalence of CTB. 

## 9. Conclusions

Cutaneous tuberculosis is a less common form of tuberculosis caused by *M. tuberculosis* that presents with various different clinical manifestations and is largely impacted by immune function. CTB can result from primary inoculation from an exogenous source through a defect in the skin barrier or from endogenous inoculation via hematogenous, lymphatic, or contiguous spread. The immune response involves neutrophils, natural killer cells, and macrophages. Granuloma formation, mediated by proinflammatory cytokine TNF-α, helps contain the infection and limit bacterial dissemination. Diagnosis of CTB is often overlooked due to its rarity and wide range of clinical manifestations, requiring a high level of suspicion and subsequent confirmation through histopathology, culture, and PCR. The histology of CTB is similar to that of *M. tuberculosis* as it exhibits characteristic inflammation, necrosis, and acid-fast bacilli. Each specific lesion may differ with regard to types of necrosis, immune cells present, and affected layers of the skin. Primary treatment of CTB involves a systemic drug regimen with an initial intensive phase utilizing isoniazid, rifampicin, and pyrazinamide. A second continuation phase is then implemented to destroy drug-resistant bacilli that may become dormant during the initial phase. Skin biopsy and subsequent acid-fast staining and cultures are used to diagnose CTB. The relation between latent TB infection and CTB has yet to be recognized but may be examined via the methodology proposed in the study by Alvarez [60]. Overall, knowledge about CTB is relatively limited, and further research is needed to explore the disease pathogenesis, risk factors, prevention, clinical testing, and treatment therapies.

## Data Availability

Not applicable.

## References

[1] van Zyl L., du Plessis J., Viljoen J. (2015). Cutaneous tuberculosis overview and current treatment regimens. Tuberculosis.

[2] Santos J.B.D., Figueiredo A.R., Ferraz C.E., Oliveira M.H.D., Silva P.G.D., Medeiros V.L.S.D. (2014). Cutaneous tuberculosis: Epidemiologic, etiopathogenic and clinical aspects-Part I. An. Bras. Dermatol..

[3] WHO (2023). Tuberculosis.

[4] Sulis G., Roggi A., Matteelli A., Raviglione M.C. (2014). Tuberculosis: Epidemiology and Control. Mediterr. J. Hematol. Infect. Dis..

[5] Mehta J.B., Dutt A.K. (2016). Epidemiology and Host Factors. Microbiol. Spectr..

[6] Hill M.K., Sanders C.V. (2017). Cutaneous Tuberculosis. Microbiol. Spectr..

[7] Sehcal V.N., Wagh S.A. (1990). Cutaneous Tuberculosis. Int. J. Dermatol..

[8] Sehgal V.N., Jain M.K., Srivastava G. (1989). Changing Pattern of Cutaneous Tuberculosis. Int. J. Dermatol..

[9] BayBay H., Senhaji I., Zinoun S., Elloudi S., Douhi Z., Mernissi F. (2021). Cutaneous tuberculosis in children from the northeastern region of Morocco. Arch. De Pédiatrie.

[10] Abdelmalek R., Mebazaa A., Berriche A., Kilani B., Ben Osman A., Mokni M., Benaissa H.T. (2013). Cutaneous tuberculosis in Tunisia. Médecine Et Mal. Infect..

[11] Vashisht P., Sahoo B., Khurana N., Reddy B. (2007). Cutaneous tuberculosis in children and adolescents: A clinicohistological study. J. Eur. Acad. Dermatol. Venereol..

[12] Ramesh V., Misra R.S., Beena K.R., Mukherjee A. (1999). A Study of Cutaneous Tuberculosis in Children. Pediatr. Dermatol..

[13] Umapathy K.C., Begum R., Ravichandran G., Rehman F., Paramasivan C.N., Ramanathan V.D. (2007). Letter to the editors. Trop. Med. Int. Health.

[14] Singal A., Sonthalia S. (2010). Cutaneous tuberculosis in children: The Indian perspective. Indian J. Dermatol. Venereol. Leprol..

[15] Chong L.-Y., Lo K.-K. (1995). Cutaneous Tuberculosis in Hong Kong: A 10-Year Retrospective Study. Int. J. Dermatol..

[16] Lotte A., Wasz-Hockert O., Poisson N., Engbaek H., Landmann H., Quast U., Andrasofszky B., Lugosi L., Vadasz I., Mihailescu P. (1988). Second IUATLD study on complications induced by intradermal BCG-vaccination. Bull. Int. Union Against Tuberc. Lung Dis..

[17] Dhar S., Ganjoo S., Dhar S., Srinivas S.M. (2020). Disseminated cutaneous tuberculosis from BCG vaccination site in an immunocompetent child. Pediatr. Dermatol..

[18] Franco-Paredes C., Marcos L.A., Henao-Martínez A.F., Rodríguez-Morales A.J., Villamil-Gómez W.E., Gotuzzo E., Bonifaz A. (2018). Cutaneous Mycobacterial Infections. Clin. Microbiol. Rev..

[19] Barbagallo J., Tager P., Ingleton R., Hirsch R.J., Weinberg J.M. (2002). Cutaneous tuberculosis: Diagnosis and treatment. Am. J. Clin. Dermatol..

[20] Tapias L., Tapias-Vargas L.F., Tapias-Vargas L. (2008). Primary cutaneous inoculation tuberculosis in a healthcare worker as a result of a surgical accident. Int. J. Dermatol..

[21] Cousins D.V., Bastida R., Cataldi A., Quse V., Redrobe S., Dow S., Duignan P., Murray A., Dupont C., Ahmed N. (2003). Tuberculosis in seals caused by a novel member of the Mycobacterium tuberculosis complex: *Mycobacterium pinnipedii* sp. nov. Int. J. Syst. Evol. Microbiol..

[22] Okazaki T., Ebihara S., Takahashi H., Asada M., Sato A., Seki M., Ohto H., Sasaki H. (2005). Multiplex PCR-Identified Cutaneous Tuberculosis Evoked by *Mycobacterium bovis* BCG Vaccination in a Healthy Baby. J. Clin. Microbiol..

[23] Sibandze D.B., Magazi B.T., Malinga L.A., Maningi N.E., Shey B.-A., Pasipanodya J.G., Mbelle N.N. (2020). Machine learning reveals that Mycobacterium tuberculosis genotypes and anatomic disease site impacts drug resistance and disease transmission among patients with proven extra-pulmonary tuberculosis. BMC Infect. Dis..

[24] Mei Y.-M., Zhang W.-Y., Sun J.-Y., Jiang H.-Q., Shi Y., Xiong J.-S., Wang L., Chen Y.-Q., Long S.-Y., Pan C. (2023). Genomic characteristics of Mycobacterium tuberculosis isolates of cutaneous tuberculosis. Front. Microbiol..

[25] Dharmadhikari A.S., Nardell E.A. (2008). What Animal Models Teach Humans about Tuberculosis. Am. J. Respir. Cell Mol. Biol..

[26] Sun H., Ma X., Zhang G., Luo Y., Tang K., Lin X., Yu H., Zhang Y., Zhu B. (2012). Effects of immunomodulators on liquefaction and ulceration in the rabbit skin model of tuberculosis. Tuberculosis.

[27] Zhan L., Tang J., Sun M., Qin C. (2017). Animal Models for Tuberculosis in Translational and Precision Medicine. Front. Microbiol..

[28] Lurie M.B. (1964). Resistance to Tuberculosis: Experimental Studies in Native and Acquired Defensive Mechanism.

[29] Zhang G., Zhu B., Shi W., Wang M., Da Z., Zhang Y. (2010). Evaluation of mycobacterial virulence using rabbit skin liquefaction model. Virulence.

[30] Zhan L., Ding H., Lin S., Tang J., Deng W., Xu Y., Xu Y., Qin C. (2014). Experimental *Mycobacterium tuberculosis* infection in the Chinese tree shrew. FEMS Microbiol. Lett..

[31] Chen Q., Chen W., Hao F. (2019). Cutaneous tuberculosis: A great imitator. Clin. Dermatol..

[32] Meier T., Eulenbruch H.-P., Wrighton-Smith P., Enders G., Regnath T. (2005). Sensitivity of a new commercial enzyme-linked immunospot assay (T SPOT-TB) for diagnosis of tuberculosis in clinical practice. Eur. J. Clin. Microbiol. Infect. Dis..

[33] Skjøt R.L.V., Oettinger T., Rosenkrands I., Ravn P., Brock I., Jacobsen S., Andersen P. (2000). Comparative Evaluation of Low-Molecular-Mass Proteins from *Mycobacterium tuberculosis* Identifies Members of the ESAT-6 Family as Immunodominant T-Cell Antigens. Infect. Immun..

[34] Pathan A.A., Wilkinson K.A., Klenerman P., McShane H., Davidson R.N., Pasvol G., Hill A.V.S., Lalvani A. (2001). Direct Ex Vivo Analysis of Antigen-Specific IFN-γ-Secreting CD4 T Cells in *Mycobacterium tuberculosis*-Infected Individuals: Associations with Clinical Disease State and Effect of Treatment. J. Immunol..

[35] Pollock J.M., Andersen P. (1997). The Potential of the ESAT-6 Antigen Secreted by Virulent Mycobacteria for Specific Diagnosis of Tuberculosis. J. Infect. Dis..

[36] Ravn P., Munk M.E., Andersen A.B., Lundgren B., Lundgren J.D., Nielsen L.N., Kok-Jensen A., Andersen P., Weldingh K. (2005). Prospective Evaluation of a Whole-Blood Test Using *Mycobacterium tuberculosis* -Specific Antigens ESAT-6 and CFP-10 for Diagnosis of Active Tuberculosis. Clin. Vaccine Immunol..

[37] Almaguer-Chávez J., Ocampo-Candiani J., Rendón A. (2009). Current Panorama in the Diagnosis of Cutaneous Tuberculosis. Actas Dermo-Sifiliográficas.

[38] Dias M.F.R.G., Filho F.B., Quaresma M.V., Nascimento L.V.D., Nery J.A.D.C., Azulay D.R. (2014). Update on cutaneous tuberculosis. An. Bras. de Dermatol..

[39] Beretta-Piccoli B.T., Mainetti C., Peeters M.-A., Laffitte E. (2018). Cutaneous Granulomatosis: A Comprehensive Review. Clin. Rev. Allergy Immunol..

[40] Lai-Cheong J.E., Perez A., Tang V., Martinez A., Hill V., Menagé H.d.P. (2007). Cutaneous manifestations of tuberculosis. Clin. Exp. Dermatol..

[41] Guirado E., Schlesinger L.S., Kaplan G. (2013). Macrophages in tuberculosis: Friend or foe. Semin. Immunopathol..

[42] Da Silva D.A.A., Da Silva M.V., Barros C.C.O., Alexandre P.B.D., Timóteo R.P., Catarino J.S., Sales-Campos H., Machado J.R., Rodrigues D.B.R., Oliveira C.J. (2018). TNF-α blockade impairs in vitro tuberculous granuloma formation and down modulate Th1, Th17 and Treg cytokines. PLoS ONE.

[43] Kaul S., Kaur I., Mehta S., Singal A. (2022). Cutaneous tuberculosis. Part I: Pathogenesis, classification, and clinical features. J. Am. Acad. Dermatol..

[44] Bravo F.G., Gotuzzo E. (2007). Cutaneous tuberculosis. Clin. Dermatol..

[45] Macgregor R.R. (1995). Cutaneous tuberculosis. Clin. Dermatol..

[46] De Brito A.C., de Oliveira C.M.M., Unger D.A.-A., Bittencourt M.D.J.S. (2022). Cutaneous tuberculosis: Epidemiological, clinical, diagnostic and therapeutic update. An. Bras. de Dermatol..

[47] Umapathy K.C., Begum R., Ravichandran G., Rahman F., Paramasivan C.N., Ramanathan V.D. (2006). Comprehensive findings on clinical, bacteriological, histopathological and therapeutic aspects of cutaneous tuberculosis. Trop. Med. Int. Health.

[48] Kim G.-W., Park H.-J., Kim H.-S., Kim S.-H., Ko H.-C., Kim B.-S., Kim M.-B., Sim E.-K. (2012). Delayed Diagnosis of Scrofuloderma Misdiagnosed as a Bacterial Abscess. Ann. Dermatol..

[49] Mann D., Sant’anna F.M., Schmaltz C.A.S., Rolla V., Freitas D.F.S., Lyra M.R., Sampaio F.M.S., Valle A.C.F., Msc M.C.S.L., Quintella L.P. (2019). Cutaneous tuberculosis in Rio de Janeiro, Brazil: Description of a series of 75 cases. Int. J. Dermatol..

[50] Wankhade V.H., Supekar B.B., Singh R.P., Ghanate T.D., Bhat D. (2021). Clinical spectrum of cutaneous tuberculosis in Central India: A retrospective study. Indian Dermatol. Online J..

[51] Zhang J., Fan Y., Wang P., Chen Q., Wang G., Xu A., Chen L., Hu R., Chen W., Song Z. (2018). Cutaneous tuberculosis in China—A multicentre retrospective study of cases diagnosed between 1957 and 2013. J. Eur. Acad. Dermatol. Venereol..

[52] Stratman S., Diaz-Perez J., Romanelli P., Lev-Tov H. (2021). Development of Chancre-Variant Cutaneous Tuberculosis After BCG Vaccine Administration in a Patient With Migraines. Wounds A Compend. Clin. Res. Pr..

[53] Keijsers R.R.M.C., Bovenschen H.J., Seyger M.M.B. (2010). Cutaneous complication after BCG vaccination: Case report and review of the literature. J. Dermatol. Treat..

[54] Ahmad C., Rupinder M., Amanda M. (2022). Oakley, ‘Cutaneous Tuberculosis’, StatPearls, Jan. https://www.ncbi.nlm.nih.gov/books/NBK482220/#:~:text=Treatment%20of%20cutaneous%20tuberculosis%20is,pyrazinamide%2C%20and%20ethambutol%20or%20streptomycin.

[55] Joshi J.M. (2011). Tuberculosis Chemotherapy in the 21st Century: Back to the Basics. Lung India.

[56] Tehrany Y.A., Toutous-Trellu L., Trombert V., Reny J.-L., Kaya G., Prendki V. (2015). A Case of Tuberculous Granulomatous Panniculitis without Vasculitis. Case Rep. Dermatol..

[57] Mei X., Zhao J. (2017). Successful treatment of erythema induratum with topical application of antituberculous drugs. Medicine.

[58] Charifa A., Mangat R., Oakley A.M. (2023). Cutaneous Tuberculosis.

[59] Khadka P., Koirala S., Thapaliya J. (2018). Cutaneous Tuberculosis: Clinicopathologic Arrays and Diagnostic Challenges. Dermatol. Res. Pr..

[60] Alvarez A.H. (2021). Revisiting tuberculosis screening: An insight to complementary diagnosis and prospective molecular approaches for the recognition of the dormant TB infection in human and cattle hosts. Microbiol. Res..

